# Reoptimization of parameterized problems

**DOI:** 10.1007/s00236-022-00428-y

**Published:** 2022-07-25

**Authors:** Hans-Joachim Böckenhauer, Elisabet Burjons, Martin Raszyk, Peter Rossmanith

**Affiliations:** 1 Department of Computer Science, ETH Zurich, Universitätsstrasse 6, Zurich, 8092 Switzerland; 2 Department of Computer Science, RWTH Aachen, Ahornstrasse 55, Aachen, 52074 NRW Germany

## Abstract

Parameterized complexity allows us to analyze the time complexity of problems with respect to a natural parameter depending on the problem. Reoptimization looks for solutions or approximations for problem instances when given solutions to neighboring instances. We combine both techniques, in order to better classify the complexity of problems in the parameterized setting. Specifically, we see that some problems in the class of compositional problems, which do not have polynomial kernels under standard complexity-theoretic assumptions, do have polynomial kernels under the reoptimization model for some local modifications. We also observe that, for some other local modifications, these same problems do not have polynomial kernels unless $\mathbf{NP}\subseteq \mathbf{coNP/poly}$. We find examples of compositional problems, whose reoptimization versions do not have polynomial kernels under any of the considered local modifications. Finally, in another negative result, we prove that the reoptimization version of Connected Vertex Cover does not have a polynomial kernel unless Set Cover has a polynomial compression. In a different direction, looking at problems with polynomial kernels, we find that the reoptimization version of Vertex Cover has a polynomial kernel of size $\varvec{2k+1}$ using crown decompositions only, which improves the size of the kernel achievable with this technique in the classic problem.

## Introduction

In this paper, we try to combine the techniques of reoptimization and parametrization in order to have a better understanding of what makes a problem hard from a parameterized complexity point of view. The goal is, given a solution for an instance of a parameterized problem, try to look at local modifications and see if the problem becomes easier or if it stays in the same complexity class. For this, we look at classical problems in parameterized complexity, whose complexity is well understood and classified.

While the connections between reoptimization and parameterization were not systematically explored up to now, some links were already discovered. The technique of iterative compression which was introduced by Reed, Smith, and Vetta [29] was very successfully used to design parameterized algorithms, see the textbook by Cygan et al. [12] for an overview. It is closely related to common design techniques for reoptimization algorithms. Abu-Khzam et al. [1] looked at the parameterized complexity of dynamic, reoptimization-related versions of dominating set and other problems, albeit more related to a slightly different model of reoptimization as introduced by Shachnai et al. [30]. Very recently, Alman, Mnich, and Williams [2] and also Krithika et al. [24] considered dynamic parameterized problems, which can be seen as a generalization of reoptimization problems.

We start by introducing the main concepts of parameterized complexity and reoptimization that we are going to use in our results.

### Parameterized complexity

Classical complexity theory classifies problems by the amount of time or space that is required by algorithms solving them. Usually, the time or space in these problems is measured by the input size. However, measuring complexity only in terms of the input size ignores any structural information about the input instances, making problems appear sometimes more difficult than they actually are.

Parameterized complexity was developed by Downey and Fellows in a series of articles in the early 1990s [15, 16]. Parameterized complexity theory provides a theory of intractability and of fixed-parameter tractability that relaxes the classical notion of tractability, namely polynomial-time computability, by allowing non-polynomial computations only depending on a parameter independent of the instance size. For a deeper introduction to parameterized complexity, we refer the reader to Downey et al. [17, 20].

We now introduce the formal framework for parameterized complexity that we use throughout the paper. Let $$\Sigma $$ denote a finite alphabet and $${\mathbb {N}}$$ the set of natural numbers. A *decision problem* $$L $$ is a subset of $$\Sigma ^*$$. We will call the strings $$x\in \Sigma ^*$$, *input* of $$L $$, regardless of whether $$x\in L $$. A *parameterized problem* is a subset $$L \subseteq \Sigma ^* \times {\mathbb {N}}$$. An input (*x*, *k*) to a parameterized language consists of two parts where the second part is the *parameter*. A parameterized problem $$L$$ is *fixed-parameter tractable* if there exists an algorithm that given an input $$(x,k) \in \Sigma ^*\times {\mathbb {N}}$$ decides whether $$(x,k)\in L $$ in *f*(*k*)*p*(*n*) time, where *f* is an arbitrarily computable function solely in *k* and *p* is a polynomial in the total input length $$n=|x|+k$$. FPT is the class of parameterized problems which are fixed-parameter tractable.

A *kernelization* for a parameterized problem $L \subseteq \Sigma ^* \times {\mathbb {N}}$ is an algorithm that, given $(x,k) \in \Sigma ^*\times {\mathbb {N}}$, outputs in *p*(*n*) time a pair $(x',k')\in \Sigma ^* \times {\mathbb {N}}$, namely a *kernel*, such that $(x,k)\in L \iff (x',k')\in L$ and $|x'|,k'\le f(k)$, where *p* is a polynomial and *f* an arbitrary computable function; *f* is referred to as the *size* of the kernel. If for a problem $L$, the size of the kernel *f* is polynomial in *k*, we say that $L$ has a *polynomial kernel*. PK is the class of parameterized problems which have polynomial kernels.

A *Turing kernelization* is a procedure consisting of two parameterized problems $L _1$ and $L _2$ (typically $L _1=L _2$) and a polynomial *g* together with an oracle for $L _2$, such that, on an input $(x,k)\in \Sigma ^*\times {\mathbb {N}}$, the procedure outputs the answer whether $x\in L _1$ in polynomial time by querying the oracle for $L _2$ with questions of the form “Is $(x_2,k_2)\in L _2$?” for $|x_2|,k_2\le g(k)$. Essentially, a Turing kernelization allows us to use an oracle for small instances, in order to solve $L _1$ on a larger instance (*x*, *k*). A *polynomial Turing kernelization* is a Turing kernelization where *g* is a polynomial function. PTK is the class of parameterized problems which have polynomial Turing kernelizations.

The problem classes we defined up to now satisfy $\mathrm{PK}\subseteq \mathrm{PTK}\subseteq \mathrm{FPT}$. There are well-known problems; however, that are not known to be FPT. For example, *k*-Clique, which is the problem of identifying whether a graph *G* contains a clique of size *k*, is not contained in FPT under some standard complexity-theoretic assumptions. Neither is the complementary problem *k*-Independent Set, which is the problem of identifying whether a graph *G* contains an independent set of size *k*, or the *k*-Set Cover problem, where given a universe set ${U}$ and a family ${\mathcal {F}}$ of subsets of ${U}$, we are asked to determine whether there is a subset of ${\mathcal {F}}$ of size *k* which contains every element of ${U}$. For these problems outside FPT, there is a further classification of their hardness in terms of the so-called *W-hierarchy* consisting of classes *W*[*t*] for $t\in {\mathbb {N}}$, such that $W[t]\subseteq W[t+1]$. Moreover, $\mathrm{FPT}\subseteq W[1]$. For the definition of these classes and the theory behind, it we refer the reader to the reference book [17]. In this paper, we will only use the classes *W*[1] and *W*[2]. For them, we have the following characterizations in terms of complete problems: *k*-Clique and *k*-Independent Set are complete for *W*[1], and *k*-Set Cover is complete for *W*[2].

### Reoptimization

Often, one has to solve multiple instances of one optimization problem which might be somehow related. Consider the example of a timetable for some railway network. Assume that we have spent a lot of effort and resources to compute an optimal or near-optimal timetable satisfying all given requirements. Now, a small local change occurs like, e. g., the closing of a station due to construction work. This leads to a new instance of our timetable problem that is closely related to the old one. Such a situation naturally raises the question whether it is necessary to compute a new solution from scratch or whether the known old solution can be of any help. The framework of *reoptimization* tries to address this question: We are given an optimal or nearly optimal solution to some instance of a hard optimization problem, then a small local change is applied to the instance, and we ask whether we can use the knowledge of the old solution to facilitate computing a reasonable solution for the locally modified instance. It turns out that, for different problems and different kinds of local modifications, the answer to this question might be completely different. Generally speaking, we should not expect that solving the problem on the modified instance optimally can be done in polynomial time, but, in some cases, the approximability might improve a lot.

This notion of reoptimization was mentioned for the first time by Schäffter [31] in the context of a scheduling problem. Archetti et al. [4] used it for designing an approximation algorithm for the metric traveling salesman problem ($\Delta$TSP) with an improved running time, but still the same approximation ratio as for the original problem. But the real power of the reoptimization concept lies in its potential to improve the approximation ratio compared to the original problem. This was observed for the first time by Böckenhauer et al. [6] for the $\Delta$TSP, considering the change of one edge weight as a local modification. Independently at the same time, Ausiello et al. [5] proved similar results for TSP reoptimization under the local modification of adding or removing vertices.

Intuitively, the additional information that is given in a reoptimization setup seems to be rather powerful. Intriguingly, many reoptimization variants of $\textsf {NP}$-hard optimization problems are also $\textsf {NP}$-hard. A general approach toward proving the $\textsf {NP}$-hardness of reoptimization problems uses a sequence of reductions and can on a high level be described as follows [7]: Consider an $\textsf {NP}$-hard optimization problem $L$, a local modification $\mathrm {lm}$, and a resulting reoptimization problem $\mathrm {lm}$-$L$. Moreover, suppose we are able to transform an efficiently solvable instance $x'$ of $L$ to any instance *x* of $L$ in a polynomial number of local modifications of type $\mathrm {lm}$. Then, any efficient algorithm for $\mathrm {lm}$-$L$ could be used to efficiently solve $L$; thus, the NP-hardness of $L$ implies the hardness of $\mathrm {lm}$-$L$.

### Reoptimization of parameterized problems

Now that we have seen the main concepts of parameterized complexity and reoptimization, we will formally define an instance for a reoptimization parameterized problem $\mathrm {lm}$-$L$.

Given a parameterized problem $$L $$, we say that a parameterized instance (*x*, *k*) has a solution if $$(x,k)\in L $$.

If an instance $(x,k)\not \in L$, we will say that the instance does not have a solution and denote it with $\bot$. A *solution* *s* for a problem instance (*x*, *k*) is a witness of size $|s|\le p(|x|)$ for some polynomial *p*, with which we can check in polynomial time that $(x,k)\in L$. Observe that there may be more than one solution for a given instance. In order to measure how good a solution is, we have to define the *cost* of the solution. The cost function assigns an integer value to every solution. For some parameterized problems, the parameter is already a measure of the goodness of the solution. For these problems, a solution *s* has *cost* *k* if it is a solution for $(x,k)\in L$ but not a solution for $(x,k')$ for any $k'<k$, if $L$ is a minimization problem, and $k'>k$, if $L$ is a maximization problem.

In problems where the parameter *k* is an intrinsic value of the instance rather than a quality measure, we have to define an extra parameter $\gamma$ measuring the quality of the solutions. A cost function ${\mathrm{cost}} (\cdot )$ is a polynomially computable function that, given a solution *s* to an instance (*x*, *k*), computes the value of $\gamma$ corresponding to this solution. Often, this parameter will be the size of the solution, but other parameters can be used. In these problems, we redefine an instance to be a triple $(x,k,\gamma )$ where $(x,k,\gamma )\in L$ if and only if $(x,k)\in L$ and there exists a solution *s* with ${\mathrm{cost}} (s)\le \gamma$ if $L$ is a minimization problem and ${\mathrm{cost}} (s) \ge \gamma$ if $L$ is a maximization problem.

From now on, we assume that *k* is a cost parameter unless otherwise specified, and thus, we refer to instances as pairs (*x*, *k*). An *instance* of a reoptimization problem $$\mathrm {lm}$$-$$L$$ consists of: an instance of the parameterized problem $$L $$, $$(x,k)\in \Sigma ^*\times {\mathbb {N}}$$, together with a solution *s* with $${\mathrm{cost}} (s)\le k$$ for minimization problems and $${\mathrm{cost}} (s)\ge k$$ for maximization problems if it exists, i. e., if $$(x,k)\in L $$, or $$\bot $$ if $$(x,k)\notin L $$, and a locally modified instance $$(x_{\mathrm {lm}},k')$$, where $$k'= f(k)$$ for a computable function *f*. We say that $$((x,k),s,(x_{\mathrm {lm}},k'))\in \mathrm {lm} \text {-}L $$ if and only if $$(x_{\mathrm {lm}},k')\in L $$.

We will also define a *polynomial reoptimization kernel* for a reoptimization instance $((x,k),s,(x_{\mathrm {lm}},k'))$ as a polynomial kernel for $(x_{\mathrm {lm}},k')$. This makes sense because $((x,k),s,(x_{\mathrm {lm}},k'))\in \mathrm {lm} \text {-}L$ if and only if $(x_{\mathrm {lm}},k')\in L$.

### Our contribution

In this paper, we use reoptimization techniques to solve parameterized problems or to compute better kernels for them. In particular, we show in Sect. 2 that some compositional parameterized problems [8], which do not have polynomial kernels under standard complexity-theoretic assumptions, do have polynomial reoptimization kernels for some local modifications. Moreover, in Sect. 3, we show that, under the opposite local modifications, those same problems do not admit polynomial reoptimization kernels unless $\mathrm{NP}\subseteq \mathrm{coNP}/\mathrm{poly}$. We also show that some compositional problems do not have polynomial reoptimization kernels under any of the standard local modifications for graph problems, i. e., vertex or edge addition or deletion.

Section 4 contains a reduction from Set Cover parameterized by the size of the universe to Connected Vertex Cover that shows that the reoptimization of Connected Vertex Cover under edge addition does not have a polynomial reoptimization kernel unless $\mathrm{NP}\subseteq \mathrm{coNP}/\mathrm{poly}$.

We then show in Sect. 5 that, for the reoptimization version of the vertex cover problem with edge addition, the crown decomposition technique yields a reoptimization kernel of size $2k+1$.

## Kernels for compositional problems

Bodlaender et al. [8] define the concept of *compositional parameterized problems*, specifically OR-compositional and AND-compositional problems, for both of which no polynomial kernel exists under standard complexity-theoretic assumptions. In this section, we see that some of these problems do indeed have polynomial reoptimization kernels, where an optimal solution or a polynomial kernel is given for a locally modified instance.

### Preliminaries

A characterization of OR-compositional graph problems is the following.

#### Definition 1

[8] Let $$L $$ be a parameterized graph problem. If for any pair of graphs $$G_1$$ and $$G_2$$, and any integer $$k\in {\mathbb {N}}$$, we have

$$\begin{aligned}(G_1,k)\in L \vee (G_2,k)\in L \iff (G_1\cup G_2,k)\in L,\end{aligned}$$

where $$G_1\cup G_2$$ is the disjoint union of $$G_1$$ and $$G_2$$, then $$L $$ is *OR-compositional*.

Now, if we define the complement of a problem, an analogous characterization can be defined that will identify problems whose complement is OR-compositional, the so-called AND-compositional problems.

#### Definition 2

Let $$L $$ be a parameterized decision problem. The *complement* $${\bar{L}}$$ *of* $$L $$ is the decision problem resulting from reverting the yes- and no-answers.

#### Definition 3

[8] Let $$L $$ be a parameterized graph problem. If for any pair of graphs $$G_1$$ and $$G_2$$, and any integer $$k\in {\mathbb {N}}$$, we have

$$\begin{aligned}(G_1,k)\in L \wedge (G_2,k)\in L \iff (G_1\cup G_2,k)\in L,\end{aligned}$$

where $$G_1\cup G_2$$ is the disjoint union of $$G_1$$ and $$G_2$$, then $${\bar{L}}$$ is OR-compositional and $$L $$ is *AND-compositional*.

Bodlaender et al. [8] showed the following result.

#### Theorem 1

[8] NP-hard OR-compositional problems do not have polynomial kernels, unless $\mathrm{NP}\subseteq \mathrm{coNP}/\mathrm{poly}$, i. e., the polynomial hierarchy collapses.

Moreover, Drucker [18] was able to show the following.

#### Theorem 2

[18] Unless $\mathrm{NP}\subseteq \mathrm{coNP}/\mathrm{poly}$, NP-hard AND-compositional problems do not have polynomial kernels.

We prove in this section that reoptimization versions of some OR-compositional or AND-compositional problems have polynomial reoptimization kernels. Let us see now which local modifications will provide these results.

When we talk about graph problems in a reoptimization setting, four local modifications come to mind immediately, namely edge addition and deletion, and vertex addition and deletion. We now define them formally.

Given a graph $$G=(V,E)$$, and a pair of non-neighboring vertices $$u,v\in V$$, we denote an *edge addition* $$(V,E\cup \{u,v\})$$ by $$G+\{u,v\}$$, or $$G+e$$ where $$e=\{u,v\}$$. Analogously, for *edge deletion*, given an edge $$e\in E$$, $$G-e$$ is the graph $$(V,E-\{ e\})$$. Furthermore, for *vertex deletion*, given a vertex $$v\in V$$, $$G-v$$ is the subgraph induced by $$V-\{v\}$$, i. e., $$(V-\{ v\}, E')$$ where $$E'$$ is *E* without the edges incident to *v*. Finally, in the case of *vertex addition*, given a new vertex *v* and a set of edges $$E'\subseteq \bigcup _{u\in V}\{u,v\}$$, $$G+v$$ is $$G=(V\cup \{v\}, E\cup E')$$. Given a graph problem $$L $$, we call the reoptimization version of $$L $$ under edge addition, edge deletion, vertex addition, and vertex deletion $$e^+ $$-$$L $$, $$e^- $$-$$L $$, $$v^+ $$-$$L $$, and $$v^- $$-$$L $$, respectively.

We now give an example of a OR-compositional FPT problem that admits polynomial reoptimization kernels under edge addition. We want to see which are the conditions that allow us to find a kernel in this setting.

### Internal vertex subtree

A *subtree* *T* of a graph *G* is a (not necessarily induced) subgraph of *G* which is also a tree. The vertices of a tree can be classified into two categories: *leaves* are vertices of degree 1 and *internal* vertices are vertices of higher degree. Let us consider the following parameterized decision problem called the Internal Vertex SubTree problem. Given a graph *G* and an integer *k*, we have to determine whether *G* contains a subtree with at least *k* internal vertices.

The connected version of this problem, where we consider as input only pairs (*G*, *k*) where *G* is connected, is called Maximum Internal Spanning Tree and has a polynomial kernel of size 3*k* using the crown lemma [21] and an improved polynomial kernel of size 2*k* [25]. However, the general version of this problem does not have a polynomial kernel, unless $\mathrm{NP}\subseteq \mathrm{coNP}/\mathrm{poly}$. Let us see this.

#### Theorem 3

Internal Vertex SubTree in general graphs does not have a polynomial kernel unless $\mathrm{NP}\subseteq \mathrm{coNP}/\mathrm{poly}$.

#### Proof

Observe first that Internal Vertex SubTree is OR-compositional. As required by Definition 1, given two connected graphs $G_1$ and $G_2$, if one of them has a subtree with *k* internal vertices, then the disjoint union of them, i. e., the graph with two connected components $G_1$ and $G_2$ will also have one, the same one that was in $G_1$ or $G_2$. This argument easily extends to arbitrary graphs $G_1$ and $G_2$. As for the reverse implication, if a graph *G* contains two connected components $G=G_1\cup G_2$ and has such a subtree, then the whole subtree, which is connected, must be contained in one of the components, meaning that either $(G_1,k)\in \textsc {Internal Vertex SubTree}$ or $(G_2,k)\in \textsc {Internal Vertex SubTree}$.

Moreover, Internal Vertex SubTree is NP-complete (in particular NP-hard). This is because there is a straightforward reduction from Hamiltonian path (see [28]), which is well known to be NP-complete.

Finally, we see that, by Theorem 1, Internal Vertex SubTree does not have a polynomial kernel unless $\mathrm{NP}\subseteq \mathrm{coNP}/\mathrm{poly}$. $\square$

Now, we are going to prove that $e^+$-Internal Vertex SubTree has a polynomial reoptimization kernel.

#### Theorem 4

$e^+$-Internal Vertex SubTree has a polynomial reoptimization kernel of size 2*k*.

#### Proof

Let us consider an instance $((G,k),T, (G+e,k))$ for $e^+$-Internal Vertex SubTree. Recall that *T* is a subtree of *G* with at least *k* internal vertices (i. e., a solution for (*G*, *k*)) if it exists or $\bot$ otherwise. The following procedure gives a kernel of size 2*k* for the modified input $(G+e,k)$.

If *T* is a subtree with at least *k* internal vertices, then *T* is also a valid solution for $G+e$; thus, any instance where $(G,k)\in \textsc {Internal Vertex SubTree}$ implies immediately that $((G,k),T, (G+e,k))\in e^+$-Internal Vertex SubTree; thus, any trivial instance $(H,k)\in \textsc {Internal Vertex SubTree}$ of size $\le 2k$ is a kernel for $e^+$-Internal Vertex SubTree.

On the other hand, if (*G*, *k*) contains no such tree, then it suffices to check for $(G+e,k)$, whether the connected component containing the edge *e* has such a subtree because any other connected component of $G+e$ is identical to a component in *G*, and, we know that those components do not contain any subtree with at least *k* internal vertices. This means that $(G+e,k)\in \textsc {Internal Vertex SubTree}$ if and only if the connected component containing *e* has a subtree with at least *k* internal vertices. And thus, a kernel for this component is equivalent to a kernel of the whole instance. As we know that a 2*k* kernel exists for the connected case [25], we can obtain one such kernel for the connected component containing *e*; thus, we have provided a kernel of size 2*k* for $((G,k),T,(G+e,k))$. $\square$

This shows that $e^+$-Internal Vertex SubTree admits polynomial reoptimization kernels. Observe that, in this case, we would be able to find a kernel for the modified instance by using the same procedure, even if we were given only a Yes/No answer or a polynomial kernel instead of a solution for the non-modified instance. This is because given an instance (*G*, *k*) for Internal Vertex SubTree, if we are guaranteed this instance has a subtree with *k* internal vertices, then for sure $(G+e,k)$ also has one, on the other hand, if we are guaranteed that (*G*, *k*) does not have such a subtree, then if one should exist for $(G+e,k)$, it would be found in the component that contains *e*, and thus, we could build a kernel for that component. In the case, we are given just an instance (*G*, *k*) and a polynomial kernel for this instance; the way to build a kernel for $(G+e,k)$ is just to build a kernel for the component that contains *e* and give as polynomial kernel for $(G+e,k)$ the kernel obtained by taking a disjoint union of both kernels. We can find through the first kernel if (*G*, *k*) has a subtree with *k* internal vertices and in this case second kernel is not relevant; otherwise, we can look at the second kernel to determine if the component containing the edge *e* has a spanning tree with *k* internal vertices, thus solving the instance $(G+e,k)$.

### Generalization

To begin with, we observe that, in order for a problem to be solvable in an analogous way to the problem above, it is important that the property defining the problem is maintained under the local modification considered. For instance, a subtree of a graph *G* is also a subtree of the same graph with an added edge, $G+e$. However, the same does not hold for edge deletion, because the deleted edge might be part of the chosen subtree for *G*. In order to formalize this, we define the following:

#### Definition 4

(*Monotone graph problem*) A graph problem $$L $$ is called *monotone* if it is closed under removal of edges and vertices. That is, if an instance $$(G,k)\in L $$, then $$(G-e,k)\in L $$ for every $$e\in E$$ and $$(G-v,k)\in L $$ for every $$v\in V$$.

#### Definition 5

(*Comonotone graph problem*) Similarly, a graph problem $$L $$ is called *comonotone* if it is closed under addition of edges and vertices.

We see in this subsection how to construct polynomial reoptimization kernels for the reoptimization versions of some compositional graph problems that are monotone or comonotone and that are not in PK.

We realize that, in order to get similar results as in the examples above, we need the following conditions. Let $$L $$ be a graph problem;

1. $$L $$ is compositional and NP-hard.
2. Any instance of the parameterized problem $$L $$ has a polynomial kernel on the connected component of a given vertex or edge, or an instance of the rooted version of the problem $$L ^*$$ has a polynomial kernel (informally, in a rooted version of a problem, any given instance contains a distinguished vertex, the root, and the solution must contain this vertex).
3. The problem is monotone or comonotone.

The first condition ensures that the considered problem does not have a polynomial kernel unless $\mathrm{NP}\subseteq \mathrm{coNP}/\mathrm{poly}$, which makes results on reoptimization interesting. The second condition allows us to find kernels locally. The third condition allows the modification to only affect the solution locally, whereas other modifications could potentially require to look at the whole instance for a solution. Let us formalize this.

We define the *environment* of an edge *e* or a vertex *v* in a graph $G+e$ or $G+v$, as the connected component that contains *e* or *v*. For edge and vertex deletions, we say that the environment of *e* or *v* in a graph $G-e$ or $G-v$ is the connected components that are modified or generated when *e* or *v* is deleted from *G*.

Given an instance (*G*, *k*) for a parameterized problem $$L $$ on graphs whose solution can be described by a subset of vertices and edges, an instance of the rooted version $$L ^*$$ of the problem is a triplet (*G*, *v*, *k*) where $$(G,v,k)\in L ^*$$ if and only if $$(G,k)\in L $$ and there exists a solution containing *v*. We will say that a kernel $$(G',k')$$ for an instance (*G*, *k*) is a *v*-*rooted* kernel if it is a kernel for (*G*, *v*, *k*) for the rooted problem $$L ^*$$, i. e., such that $$(G',k')\in L $$ if and only if $$(G,k)\in L $$ and has a solution (represented by a subset of vertices and edges) that contains *v*.

Given a graph $G=(V,E)$, and a subset of the vertex set $V'\subseteq V$, the subgraph *H* induced by $V'$ is the graph $H=(V',E')$ where $E'\subseteq E$ contains the edges between vertices of *V* that are part of *E*.

Finally, given an instance $(G,k)\in L$ for a graph problem $L$ whose solution can be described by a subset of vertices and edges, we say that the solution $S\subseteq V$ to (*G*, *k*) is a *witness solution* if it, given the subgraph *H* induced by *S* together with a parameter, is an instance for $L$ and $(H,k)\in L$, and moreover, *S* is a solution for any supergraph $G'$ of *G* for which *H* is a subgraph induced by *S*. Essentially, we require all of the vertices which are necessary for the solution to be valid, to be part of the solution subset, and we require that the solution keeps being valid for any supergraph. This last requirement is automatically satisfied in comonotone graph problems, which would allow us to relax the definition, but is needed in the case of monotone graph problems.

We are now ready to formally state the theorems which generalize the results we have for Internal Vertex SubTree.

#### Theorem 5

Let $$L $$ be a parameterized NP-hard OR-compositional monotone graph problem. If, for every instance (*G*, *k*), we can compute a polynomial kernel for an environment of any edge $$e\in E$$ or if there exist witness solutions for instances in $$L$$ and we can compute a rooted polynomial kernel for any vertex $$v\in V$$, then $$e^-$$-$$L $$ admits polynomial reoptimization kernels. Moreover, if for every instance $$({\hat{G}},{\hat{k}})$$, we can compute a polynomial kernel for an environment of any vertex $$v\in {\hat{V}}$$, then $$v^-$$-$$L $$ admits polynomial reoptimization kernels.

In the same way, we can state a similar theorem for comonotone graph problems with the complementary reoptimization steps, namely edge and vertex addition.

#### Theorem 6

Let $$L $$ be a parameterized NP-hard OR-compositional comonotone graph problem. If, for every instance (*G*, *k*), we can compute a polynomial kernel for an environment of any edge $$e\in E$$ or if there exist witness solutions for instances in $$L$$ and we can compute a rooted polynomial kernel for any vertex $$v\in V$$, then $$e^+$$-$$L $$ admits polynomial reoptimization kernels. Moreover, if for every instance $$({\hat{G}},{\hat{k}})$$, we can compute a polynomial kernel for an environment of any vertex $$v\in {\hat{V}}$$ or a polynomial kernel rooted to *v* for any $$v\in {\hat{V}}$$; then, $$v^+$$-$$L $$ admits polynomial reoptimization kernels.

We state a proof for Theorem 5 in the case of edge deletion, and the rest of the cases can be proven by analogy to it.

#### Proof of Theorems 5 and 6

Let us first consider the case for edge deletion. If an instance (*G*, *k*) for a monotone parameterized graph problem $L$ is a yes instance then, we can construct a trivial yes-kernel for $(G-e,k)$.

Otherwise, $(G,k)\notin L$. It is important to observe, that in case of an edge deletion, given an instance *G* and an edge *e*, the environment of *e* might contain two connected components.

If the considered problem $$L $$ has polynomial kernels for an environment of an edge, it will have a polynomial kernel for the graph $$G-e$$. This is because none of the components of the rest of the graph are modified; thus, any solution found for $$G-e$$ must be in the environment of *e*, thus making the generated kernel, a valid kernel for $$(G-e,k)$$.

On the other hand, if for any instance of the considered problems we can compute a kernel for any vertex $v \in V$, in the case of edge deletion, there may be only two components in the environment of *e* after deleting it. One can thus consider the two rooted kernels on the two vertices adjacent to *e*. The union of these two kernels is a kernel for $(G-e, k)$ in this case. Let us assume that $(G-e,k)$ has a witness solution $S'$ that does not contain any of the vertices adjacent to *e*; this would mean that the subgraph *H* induced by $S'$ has *G* as a supergraph. By the definition of witness solution, if *G* is a supergraph of *H*, then $(G,k)\in L$, thus contradicting the assumption that $(G,k)\notin L$. This argument does not work in the case of vertex deletion, where the number of connected components after the deletion might not be bounded on *k*. Thus, having rooted kernels is not enough to guarantee reoptimization kernels for vertex deletion.

The cases for vertex deletion and edge and vertex addition in comonotone graph problems are completely analogous, which proves Theorems 5 and 6. $\square$

In the case of vertex deletion, the number of newly generated connected components can be as high as the degree of the deleted vertex *v*. It is important to point out that, in this case, in order to have a polynomial kernel for an environment, it might not be enough that $$L $$ is in PK if restricted to connected instances (which was true for the previous cases). This is also the reason why, if a monotone problem has a rooted kernel, the theorem still does not hold up for $$v^-$$-$$L $$, as we would need to make sure that the deleted vertex has restricted degree, too. For any vertex with degree superpolynomial in *k*, even the existence of rooted kernels for all of its neighbors would not provide us with a polynomial reoptimization kernel for $$G-v$$.

In general, polynomial kernels cannot be built for OR-compositional hard problems because one might have a lot of connected components and one can only build a polynomial kernel for each connected component. In fact, in the next section, we will see that in general some of the reoptimization versions of OR(or AND)-compositional problems do not have polynomial reoptimization kernels.

If we now come back to the Internal Vertex SubTree problem that we saw in Sect. 2.2, we realize that not only the conditions are satisfied to apply Theorem 6 for an edge addition, but also to apply it to a vertex addition. In particular, we can build kernels for the environment of any vertex. Thus, we conclude that $v^+$-Internal Vertex SubTree admits polynomial reoptimization kernels.

A problem where we can find a rooted kernel is Leaf Out Tree, known sometimes in the literature as *k*-Leaf Out Tree. Given a directed graph *D* and an integer *k*, we are asked to compute a tree in *D* with at least *k* leaves. We first observe that this problem is comonotone. Moreover, the rooted version of this problem is in PK with a quadratic kernel as was seen by Daligault and Thomassé [13]; furthermore, any subset of vertices and edges from *D* forming the tree with at least *k* leaves is a witness solution. Finally, Leaf Out Tree has no polynomial kernel unless $\mathrm{coNP}\subseteq \mathrm{NP}/\mathrm{poly}$ as pointed out by Fernau et al. [19], due to the fact that the problem is OR-compositional and NP-hard. Then, applying Theorem 6, we deduce that $e^+$-Leaf Out Tree and $v^+$-Leaf Out Tree admit polynomial reoptimization kernels.

Yet another problem that falls into this category is Clique on graphs of maximum degree *d*, where *d* is the parameter, with target solution size *k* (*d*-Clique); this problem trivially has a polynomial kernel for the rooted case, as the neighborhood of any vertex can be exhaustively checked for clique candidates, and a witness solution is simply a subset of *k* vertices that induce a clique. It is OR-compositional and does not have a polynomial kernel parameterized by maximum degree in the general case as observed by Hermelin et al. [23]; thus, we deduce that we can apply Theorem 6, and thus, $e^+$-*d*-Clique and $v^+$-*d*-Clique admit polynomial reoptimization kernels.

To sum it up, we have the following corollary:

#### Corollary 1

The following problems, parameterized by their solution size, admit polynomial reoptimization kernels:

- $v^+$-Internal Vertex SubTree
- $e^+$-Leaf Out Tree and $v^+$-Leaf Out Tree
- $e^+$-*d*-Clique and $v^+$-*d*-Clique

Now, we can think about the complementary version of the problems described, where a property is required in every component in order for a solution to exist, i. e., AND-compositional problems. Again, we can state a pair theorems for AND-compositional problems analogous to Theorems 5 and 6 based on the considered local modifications.

#### Theorem 7

Let $$L $$ be a parameterized NP-hard AND-compositional monotone graph problem. If, for every instance (*G*, *k*), we can compute a polynomial kernel for an environment of any edge $$e\in E$$, then $$e^+$$-$$L $$ admits polynomial reoptimization kernels. Moreover, if for every instance $$({\hat{G}},{\hat{k}})$$, we can compute a polynomial kernel for an environment of any vertex $$v\in {\hat{V}}$$; then, $$v^+$$-$$L $$ admits polynomial reoptimization kernels.

#### Theorem 8

Let $$L $$ be a parameterized NP-hard AND-compositional comonotone graph problem. If, for every instance (*G*, *k*), we can compute a polynomial kernel for an environment of any edge $$e\in E$$, then $$e^-$$-$$L $$ admits polynomial reoptimization kernels. Moreover, if for every instance $$({\hat{G}},{\hat{k}})$$, we can compute a polynomial kernel for an environment of any vertex $$v\in {\hat{V}}$$, then $$v^-$$-$$L $$ admits polynomial reoptimization kernels.

The proofs of these theorems are analogous to the ones for Theorems 5 and 6. We again prove the statement regarding edge addition, and the rest are proved analogously.

#### Proof

Given a solution to an instance (*G*, *k*) for a monotone problem $$L $$, we find a polynomial kernel for an instance $$(G+e,k)$$ as follows.

If $$(G,k)\notin L $$, then for sure $$(G+e,k)\notin L $$. If $$(G+e,k)$$ was in $$L $$, then any solution for $$(G+e,k)$$ would also be a solution for (*G*, *k*) because $$L $$ is monotone.

If (*G*, *k*) has a solution *S*, *S* might not be a valid solution for $$G+e$$. However, because $$L $$ is AND-compositional, it means that the required property is already satisfied in every component of $$G+e$$ except, maybe, in the environment of *e*. This means that checking whether the environment of *e* is in $$L $$ is enough to ensure that $$G-e\in L $$. Thus, a kernel for an environment of *e* will be a reoptimization kernel for the reoptimization instance.

The cases for vertex addition in monotone graph problems and edge and vertex deletion in complement of monotone graph problems are completely analogous. $\square$

Observe that the theorems for AND-compositional problems do not mention local kernels for rooted versions of the problem. This is because, when constructing a kernel for the reoptimization version of an OR-compositional problem, we are given an instance without a solution, and then, the modified instance might have a solution. Intuitively, it is clear that the new solution has to be around the local modification. In AND-compositional problems, however, the procedure is exactly the opposite. Given an instance that has a solution, we are provided with a local modification that renders that solution useless. Essentially, we need to make sure that the component or components affected by the modification still have a solution. This solution will thus not need to be a new solution, but one that might already have existed within the component in the original instance, but that was not given in the reoptimization instance, as the reoptimization instance only requires one solution for the original instance to be given.

## Reoptimization compositional problems without polynomial kernels

We have just presented a general strategy to construct polynomial reoptimization kernels for reoptimization versions of OR-compositional and AND-compositional problems. Let us focus now on proving which of these problems do not have polynomial reoptimization kernels for some other local modifications. That is, problems where even knowing an optimal solution for a neighboring instance does not help to build a kernel for the given instance.

First, we give an intuitive approach to the kernelization results for compositional problems. In order to build kernels for reoptimization versions of OR-compositional and AND-compositional problems, we took a local modification that would not break the solution, i.e., a local modification that would respect the monotonicity properties of the problem. Through this monotonicity, we then could build a kernel centered on the local modification, knowing that the rest of the solution remains valid.

Now we try to do the opposite. That is, we will take local modifications which go against the monotonicity of the problem properties. Then, we use a clever built-in solution for a neighboring instance that will be broken when the local modification occurs, yielding the knowledge of the neighboring solution useless.

In order to prove these results, we reduce parameterized problems to their reoptimization versions, in a way analogous to polynomial parameter transformations.

### Definition 6

(Bodlaender et al. [9]) Given two parameterized problems $L$ and *Q* an algorithm *A* is a *polynomial parameter transformation* from $L$ to *Q* if given an instance (*x*, *k*) for $L$, *A* transforms it in polynomial time into an instance $(x',k')$ for *Q* such that $k'$ is polynomial in *k* and $(x,k)\in L$ if and only if $(x',k')\in Q$.

We can extend this definition to be able to reduce parameterized problems to parameterized reoptimization problems.

### Definition 7

Given a parameterized problem $L$ and a reoptimization problem lm-*Q* an algorithm *A* is a *polynomial parameter transformation* from $L$ to lm-*Q* if given an instance (*x*, *k*) for $L$, *A* transforms it in polynomial time into an instance $((x',k'),s,(x'_{\mathrm{lm}},k''))$ for *Q* such that $k'$ and $k''$ are polynomial in *k* and $(x,k)\in L$ if and only if $((x',k'),s,(x'_{\mathrm{lm}},k''))\in Q$.

With this definition, we are able to reduce parameterized problems into reoptimization problems, which helps us prove lower bounds for the existence of polynomial reoptimization kernels for the latter ones. Let us observe this through an example.

### $e^-$-longest path

We now give an example of an OR-compositional FPT problem that does not have polynomial reoptimization kernels for some local modifications. We want to see which are the conditions that make finding a kernel in this setting as difficult as the original problem.

In the parameterized Longest Path problem, the goal is given an instance (*G*, *k*) to determine whether *G* contains a path of length at least *k*.

It is easy to see that this problem is OR-compositional, and it is NP-complete [10], so in general, according to Theorem 1, it is not in PK unless $\mathrm{NP}\subseteq \mathrm{coNP}/\mathrm{poly}$.

We are going to show now that this even holds for certain reoptimization variants.

#### Theorem 9

$e^-$-Longest Path and $v^-$-Longest Path do not have polynomial reoptimization kernels unless $\mathrm{NP}\subseteq \mathrm{coNP}/\mathrm{poly}$.

#### Proof

We prove the claim by providing a reduction from Longest Path to $e^-$-Longest Path through a polynomial parameter transformation.

Given an instance (*G*, *k*) for Longest Path, we construct an instance for $e^-$-Longest Path as follows. Given $P_k$ a path of length *k*, let $((G\cup P_k,k), P_k, (G\cup (P_k-e),k))$ be an instance for $e^-$-Longest Path where *e* is an edge in $P_k$. We observe, that after deleting an edge from $P_k$, $P_k$ is no longer a path of length *k* and thus $((G\cup P_k,k), P_k, (G\cup (P_k-e),k))\in e^-$-Longest Path if and only if $(G,k)\in \textsc {Longest Path}$. Moreover, the solution $P_k$ does not provide any information about the graph *G* in which the new solution must be found. Thus, if $e^-$-Longest Path would have a polynomial reoptimization kernel, given any instance (*G*, *k*) of Longest Path, we would be able to construct a kernel for it by providing a kernel for $((G\cup P_k,k), P_k, (G\cup (P_k-e),k))$. But Longest Path is not in PK unless $\mathrm{NP}\subseteq \mathrm{coNP}/\mathrm{poly}$, thus proving the statement.

The reduction for $v^-$-Longest Path is completely analogous. $\square$

The insight that this example provides is that if a reoptimization instance has an easy-to-spot solution that is not available after the reoptimization step, then solving this instance might be as hard as solving the problem in general without any extra information.

### General results

In order to prove a general result about reoptimization versions of OR- and AND-compositional problems, we need to understand what an easy solution, or an easy-to-break solution looks like.

We say that a graph *G* is *maximal* with respect to the problem $$L $$ and the parameter *k* if $$(G,k)\in L $$, but for any edge *e*, $$(G+e,k)\not \in L $$. Analogously, we say that *G* is *minimal* if $$(G,k)\in L $$, but $$(G-e,k)\not \in L $$ and $$(G-v,k)\not \in L $$ for any edge *e* or vertex *v*.

Observe that the notion of maximality does not include vertex addition. This is because, when adding vertices to a graph, there is too much freedom on how to make the new vertex adjacent to a specific subset of vertices of the original graph. In this sense, it can be considered that vertex addition is not so much of a local modification as it is a global one. In particular, when thinking about maximal graphs, there exist problems for which specific graphs are maximal only if vertices are added adjacent to all the other vertices or to none of them. Consequently, this section includes no results for vertex addition.

Maximal or minimal graphs, if easy to construct, will help us design polynomial parameter transformations from an instance for a graph problem $L$, to an instance for its reoptimization version such that the existence of polynomial reoptimization kernels would imply that $L$ is also in PK.

#### Theorem 10

Let $$L$$ be a monotone (comonotone) NP-hard OR-compositional graph problem. If, given an instance (*G*, *k*) for $$L$$, we can compute in time polynomial in *k* a maximal (minimal resp.) graph with respect to *k*, then $$e^+$$-$$L $$ ($$e^-$$-$$L $$ and $$v^-$$-$$L $$ resp.) do not admit polynomial reoptimization kernels, unless $$\mathrm{NP}\subseteq \mathrm{coNP}/\mathrm{poly}$$.

#### Proof

Let $L$ be a monotone NP hard OR-compositional graph problem and let (*G*, *k*) be an instance for $L$. Let then *H* be an maximal graph with respect to $L$ and *k*, and *S* be a solution for (*H*, *k*).

Observe that, because $$L$$ is OR-compositional, $$(H,k)\in L $$ implies that $$(G\cup H)\in L $$. Thus, the instance $$((G\cup H,k), S, (G\cup (H+e),k))$$ for $$e^+$$-$$L $$ will only be in $$e^+$$-$$L $$ if $$(G,k)\in L $$, as $$(H+e,k)\not \in L $$ by construction.

Thus, if $e^+$-$L$ would admit polynomial reoptimization kernels, given any instance (*G*, *k*) of $L$ we would be able to construct a kernel for it by providing a kernel for $((G\cup H,k), H, (G\cup (H+e),k))$. But $L$ is not in PK, unless $\mathrm{NP}\subseteq \mathrm{coNP}/\mathrm{poly}$, thus proving the statement.

An analogous construction proves the statement for problems that are comonotone. $\square$

In particular, as a corollary we have:

#### Corollary 2

The following problems, parameterized by the solution size, do not admit polynomial reoptimization kernels, unless $\mathrm{NP}\subseteq \mathrm{coNP}/\mathrm{poly}$.

- $e^-$-Internal Vertex SubTree and $v^-$-Internal Vertex SubTree
- $e^-$-Leaf Out Tree and $v^-$-Leaf Out Tree
- $e^-$-*d*-Clique and $v^-$-*d*-Clique
- $e^-$-Clique and $v^-$-Clique

#### Proof

We have proved already that Internal Vertex SubTree, Leaf Out Tree and *d*-Clique are NP-hard, OR-compositional and comonotone. Moreover, a tree with *k* internal vertices is minimal for Internal Vertex SubTree, a directed tree with *k* leaves is minimal for Leaf Out Tree and the complete graph with *k* vertices, and $K_{k}$ is minimal for *d*-Clique, all of them computable in polynomial time. For Clique it is even simpler, as *d*-Clique is a subproblem of Clique; thus, the nonexistence of polynomial kernels for reoptimization versions of *d*-Clique implies that such kernels also do not exist for Clique. $\square$

For AND-compositional graph problems, a similar result can be stated by constructing a reoptimization instance with a graph that is maximal or minimal with respect to the complement problem.

#### Theorem 11

Let $$L$$ be a monotone (comonotone) NP-hard AND-compositional graph problem. If, given an instance (*G*, *k*) for $$L$$, we can compute in time polynomial in *k* a minimal (maximal resp.) graph with respect to *k*, $$e^-$$-$$L $$ and $$v^-$$-$$L $$ ($$e^+$$-$$L $$ resp.) do not admit polynomial reoptimization kernels, unless $$\mathrm{NP}\subseteq \mathrm{coNP}/\mathrm{poly}$$.

#### Proof

Let $L$ be a monotone NP-hard AND-compositional graph problem and let (*G*, *k*) be an instance for $L$. Let then *H* be a minimal graph with respect to $L ^c$ and *k*. This means that $(H,k)\not \in L$, however, $(H-e,k) \in L$ for any edge *e*.

The instance $$((G\cup H,k), \bot , (G\cup (H-e),k))$$ for $$e^-$$-$$L $$ will only be in $$e^-$$-$$L $$ if $$(G,k)\in L $$, as $$(H-e,k)\in L $$ by construction.

Thus, if $e^-$-$L$ would admit polynomial reoptimization kernels, given any instance (*G*, *k*) of $L$ we would be able to construct a kernel for it by providing a kernel for $((G\cup H,k), \bot , (G\cup (H-e),k))$. But $L$ is not in PK, unless $\mathrm{NP}\subseteq \mathrm{coNP}/\mathrm{poly}$, thus proving the statement.

An analogous construction proves the statement for $$v^-$$-$$L $$ and for problems that are comonotone. $$\square $$

Let us now present a problem that this theorem can be applied to, the Tree Width problem. The aim of this problem is to measure how tree-like a graph is. In order to do so, we define the following structure.

#### Definition 8

Let $G=(V,E)$ be a graph. A *tree decomposition* of *G* is a pair $D=(T,B)$, where $T=(V_T,E_T)$ is a tree. Let *I* denote an arbitrary index set enumerating the vertices from $V_T$. Then, *B* is a labeling function $B:I\rightarrow 2^{V}$ that assigns a vertex set $X_i\subseteq V$ to each index $i\in I$ (that is, to each vertex from $V_T$). These sets $X_i$ are called *bags*. Moreover, *D* satisfies the following properties:

$$\begin{aligned} \bigcup _{i\in I}X_i=V, \end{aligned}$$

for every edge $\{u,v\}\in E$, there exists an index $i\in I$ such that $u,v\in X_i$, and for each $v \in V$, the bags $X_i$ containing *v* are assigned to a subtree of *T*.

The *width* of *D* is defined as $\max \{|X_i| \mid i\in I\}-1$, that is, the maximum size of a bag minus 1. The *treewidth* of *G* is the minimum width over all tree decompositions of *G*; it is denoted by $\mathrm{tw}(G)$.

Given an instance (*G*, *k*) consisting of a graph *G* and a parameter *k*, we say that $(G,k)\in \textsc {Tree Width}$ if and only if $\mathrm{tw}(G)\le k$.

This problem is NP-hard [22], AND-compositional [8], and monotone, as we can observe, by definition, that given a tree decomposition for a graph *G*, it is also a tree decomposition for any $G-e$ or $G-v$ if we remove the removed vertex from the bags containing it.

In particular, as a corollary we have:

#### Corollary 3

$e^-$-Tree Width and $v^-$-Tree Width do not admit polynomial reoptimization kernels, unless $\mathrm{NP}\subseteq \mathrm{coNP}/\mathrm{poly}$.

Let us also see a concrete proof to see how one constructs the instances mentioned in the proof of Theorem 11.

#### Proof

Let (*G*, *k*) be an instance for Tree Width. Let now *H* be a graph with treewidth $k+1$ such that, for any edge *e*, $H-e$ has treewidth *k*. For instance, the complete graph with $k+2$ vertices $K_{k+2}$ fulfills this property. If $e^-$-Tree Width admitted polynomial reoptimization kernels, it would be able to provide a polynomial kernel for the instance $((G\cup K_{k+2},k),\bot ,(G\cup (K_{k+2}-e,k))$. Observe that because $K_{k+2}$ has treewidth $k+1$, the treewidth of $G\cup K_{k+2}>k$, thus $(G\cup K_{k+2},k)\notin \textsc {Tree Width}$ and it is valid to put $\bot$ as the second element of the instance. Moreover, because $K_{k+2}-e$ has treewidth *k*, $((G\cup K_{k+2},k),\bot ,(G\cup (K_{k+2}-e,k))\in e^-$-$\textsc {Tree Width}$ if and only if $(G,k)\in \textsc {Tree Width}$; thus, a kernel for the reoptimization instance would provide us with a kernel for the initial instance. $\square$

### Other reoptimization compositional problems without polynomial kernels

We have seen that for every monotone, or comonotone, NP-hard compositional graph problem, two of its reoptimization variants do not admit polynomial reoptimization kernels. Nevertheless, finding local kernels for the other two reoptimization variants is not trivial either.

We now provide problems for which even the notion of fixed-parameter tractability cannot be transferred to the reoptimization setting. Given an instance $((x,k),s, (x_{\mathrm{lm}},k'))$ for a parameterized reoptimization problem lm-$L$, recall that $((x,k),s,(x_{\mathrm {lm}},k'))\in \mathrm {lm} \text {-}L$ if and only if $(x_{\mathrm {lm}},k')\in L$. We say that we solve an instance $((x,k),s, (x_{\mathrm{lm}},k'))$ in fixed-parameter tractable time, if and only if we can decide whether $(x_{\mathrm {lm}},k')\in L$ in time $f(k)\cdot p(n)$, for an arbitrary function *f* and a polynomial *p*.

#### Clique

We already know that reoptimization Clique instances parameterized by the size of the clique do not have polynomial reoptimization kernels in the case of edge and vertex deletion.

Recall also that Clique is conjectured not to be in FPT since it is *W*[1]-hard.

We now show that under any of the four considered local modifications the reoptimization versions of Clique are not likely to be solvable in FPT time.

##### Theorem 12

$e^-$-$\textsc {Clique}$, $v^-$-$\textsc {Clique}$, $e^+$-$\textsc {Clique}$, and $v^+$-$\textsc {Clique}$ are not solvable in fixed-parameter tractable time, unless $\mathrm{FPT}=W[1]$.

##### Proof

We start by showing this result for $e^+$-$\textsc {Clique}$ through a polynomial parameter transformation from Clique.

Let (*G*, *k*) be an instance for Clique. We construct the following instance for $e^+$-$\textsc {Clique}$. Let $G'$ be a graph that consists of the graph *G* together with $v_1$ and $v_2$, two new vertices adjacent to every vertex in *G* but not to each other. Let then $e_{1,2}$ denote the edge between $v_1$ and $v_2$. Then, $((K_{k+1}\cup G',k+1),K_{k+1},(K_{k+1}\cup G'+e_{1,2}, k+2))\in e^+$-$\textsc {Clique}$ if and only if $(G,k)\in \textsc {Clique}$. Observe, that, because Clique is a maximization problem, it is possible to make the parameter larger.

If an instance for $e^+$-Clique was solvable in fixed-parameter tractable time, it would be possible to solve any given instance of Clique in fixed-parameter tractable time because of this construction.

As for $v^+$-$\textsc {Clique}$, we just need to consider, given an instance (*G*, *k*) for Clique, the reoptimization instance $((K_k\cup G,k),K_k,(K_k\cup G + v_1,k+1))$, where $v_1$ is adjacent to every vertex in *G*, and observe again that this instance is in $v^+$-$\textsc {Clique}$ if and only if $(G,k)\in \textsc {Clique}$. Thus, we reach the same conclusion.

For the edge and vertex deletion cases, one only needs to consider the instances $((K_{k}\cup G,k),K_{k},(K_{k}-e\cup G, k))$ and $((K_{k}\cup G,k),K_{k},(K_{k}-v\cup G, k))$, where *e* and *v* are any edge or vertex in the clique of size *k*, respectively. $\square$

## Non-compositional problems without polynomial kernels: connected vertex cover

One of the non-compositional problems in which reoptimization does not help us to achieve any improvement with respect to the classical parametrization techniques is the Connected Vertex Cover (CVC) problem. A *connected vertex cover* of a graph $G=(V,E)$ is a subset of vertices $A\subseteq V$ that is a vertex cover of *G* such that the subgraph induced by *A* is connected. Connected Vertex Cover is FPT with respect to the solution size [11]. Moreover, it is conjectured that Connected Vertex Cover does not have a polynomial Turing Kernel [23].

We build a reduction from Set Cover that will show that even the reoptimization versions of Connected Vertex Cover do not have a polynomial kernel unless Set Cover has a polynomial compression with respect to its universe size. First we define the notion of polynomial compression. Informally, we can think of a compression as a way to transform an instance for a problem $L _1$ into a kernel for a problem $L _2$. This concept is a bit more general than kernelization in the sense that it allows to show non-kernelization results for problems that are NP-hard: If an NP-complete problem compresses to a problem *X*, which is also in NP, then the compressed instance of *X* can be transformed back into an instance of the original problem. Hence, a polynomial compression gives you automatically a polynomial kernel.

### Definition 9

(Cygan et al. [12]) A *polynomial compression* of a parameterized language $Q\subseteq \Sigma ^*\times {\mathbb {N}}$ into a language $R\subseteq \Sigma ^*$ is an algorithm that takes as input an instance $(x,k)\in \Sigma ^*\times {\mathbb {N}}$, works in time polynomial in $|x|+k$, and returns a string *y* such that $|y|\le p(k)$, for some polynomial *p*, and $y\in R$ if and only if $(x,k)\in Q$.

Moreover, Dom et al. [14] prove that Set Cover parameterized by the size of the universe does not have a polynomial compression, unless $\mathrm{NP}\subseteq \mathrm{coNP}/\mathrm{poly}$.

We prove through a reduction that if $e^+$-Connected Vertex Cover had a polynomial reoptimization kernel, then Set Cover parameterized by the size of the universe would have a polynomial compression. Formally:

### Theorem 13

$e^+$-Connected Vertex Cover does not have a polynomial reoptimization kernel, unless $\mathrm{NP}\subseteq \mathrm{coNP}/\mathrm{poly}$.

### Proof

We describe the polynomial parameter transformation from Set Cover parameterized by the size of the universe to Connected Vertex Cover. Then, we use this reduction to prove that if a polynomial reoptimization kernel would exist for $e^+$-Connected Vertex Cover, then we would have a polynomial compression for Set Cover parameterized by the size of the universe, but such a compression is not possible, unless $\mathrm{NP}\subseteq \mathrm{coNP}/\mathrm{poly}$.

A Set Cover instance parameterized by the size of the universe is a quadruple, $(({U},{\mathcal {F}}, k), u)$ where $({U},{\mathcal {F}}, k)$ is the instance, comprised by ${U} =\{1,\ldots , u\}$, the universe set, of size $|{U} |=u$, ${\mathcal {F}} =\{F_1,\ldots F_t\}$, a family of subsets, and *k*, the solution size targeted, and *u* is the parameter, as defined in the introduction. We want to answer the question: Is there a subfamily of *k* sets of ${\mathcal {F}}$ that covers ${U}$?

Until now, we always considered the solution size as the parameter in all of our parameterized problems. This, however, is not fixed as such in the definition of kernelization, which allows us to choose the parameter with other criteria, as we do in this case.

We only consider instances for Set Cover where $k\le u$, because otherwise the solution is trivial. This is because any subset $F_i$ in the optimal solution should cover at least one element in ${U}$ that is not covered by any other selected subset. Otherwise, the subset could be trivially removed and the solution would be smaller. Moreover, if the number of subsets in the family ${\mathcal {F}}$ is smaller than *k*, a solution can trivially include all of the subsets. Thus, in the following we assume that $t>k$.

**Fig. 1:**
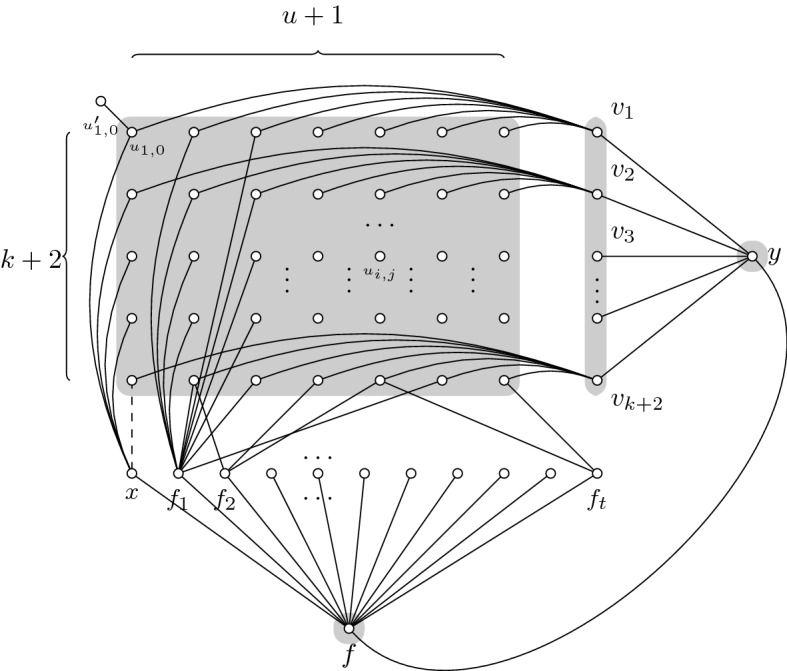
Drawing of the reduction graph. Here the leaves $u'_{i,j}$ are left out of the drawing for clarity. The connected vertex cover for this graph using the side vertices $v_m$ is shaded in gray

Let us first show the following reduction from Set Cover to Connected Vertex Cover. Given a Set Cover instance $(({U},{\mathcal {F}}, k), u)$, we construct an instance for Connected Vertex Cover as shown in Fig. 1.

We create a grid of vertices $u_{i,j}$ where $i=1,\ldots ,k+2$ and $j=0,\ldots , u$. Each of these vertices has an attached leaf $u'_{i,j}$. Each one of the columns 1 to *u* of the grid represents one of the elements of the universe set in the Set Cover instance. Column 0 is an additional column which can be viewed as an extra element added to the set.

We add a row of vertices $f_1,\ldots , f_t$ such that each vertex $u_{i,j}$ of the column $j>0$ will be connected to $f_\ell$ if and only if $j\in F_\ell$. We also add a vertex *x* which is connected to all of the vertices of the first column $u_{i,0}$ for all $i=1,\ldots ,k+1$, except for $u_{k+2,0}$. We can think of *x* as an extra set in the family of subsets ${\mathcal {F}}$, containing only the new element of the set. The edge $e=(x,u_{k+2,0})$ will be added in the reoptimization step (dashed edge in Fig. 1).

We add a column $v_1,\ldots , v_{k+2}$ such that each vertex $u_{i,j}$ of row *i*, will be connected to $v_i$, as represented in Fig. 1. Finally, we add two vertices *f* and *y*, *f* neighboring $f_\ell$, for all $\ell =1,\ldots ,t$, and also *x* and *y*, and *y* additionally neighboring $v_i$, for all $i=1,\ldots ,k+2$.

Now we will use this reduction to prove Theorem 13.

Given a Set Cover instance $(({U},{\mathcal {F}}, k), u)$ with $k< u$, we construct an $e^+$-Connected Vertex Cover instance as follows. Take first the graphs *G*, $G+e$ constructed by the reduction described above. Now, this instance is not complete unless we provide the appropriate parameters for *G* and $G+e$ and a solution for *G*.

We will now construct two optimal solutions for *G*.

To select a connected vertex cover in the graph, first observe that if the grid vertex $u_{i,j}$ is not part of the connected vertex cover, then, even if all the other vertices in the graph were in the cover, the cover would not be connected.

Thus, we select all the vertices $u_{i,j}$ of the grid (i. e., $(k+2)(u+1)$ vertices in total). This covers all leaf edges $(u_{i,j},u'_{i,j})$, all edges $(u_{i,j},f_\ell )$ and all edges $(u_{i,j},v_{i})$. It does not cover, however, the edges $(f_\ell ,f)$, $(v_{i}, y)$ and (*y*, *f*), and it is not connected.

**Fig. 2:**
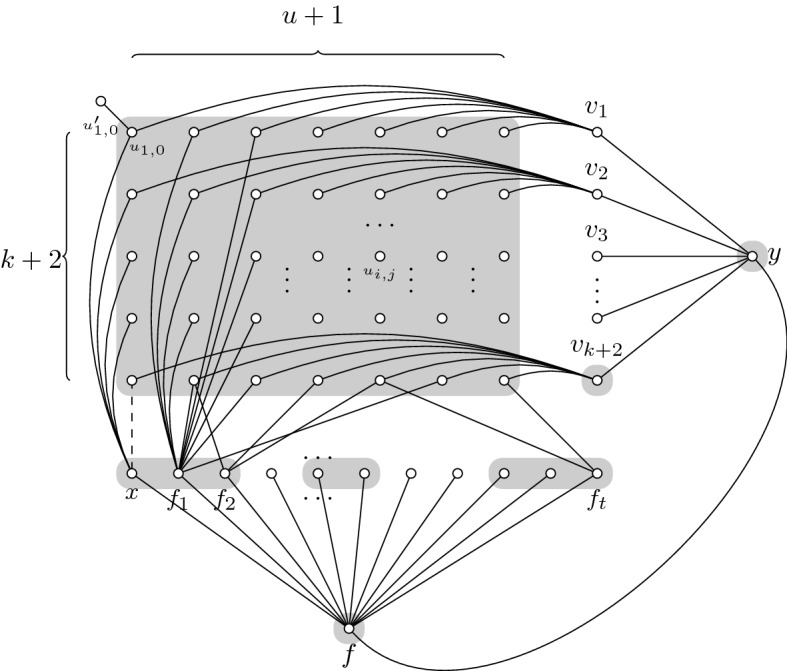
Second CVC option using a SC for ${\mathcal {F}}$. Observe, that not all of the vertices $f_i$, are part of the cover, only a selection of them

The vertex *f* needs to be taken to cover the edges $(f_\ell ,f)$ because, again, recall we assumed that $t>k$, thus taking every $f_\ell$ would make the vertex cover too large with respect to *u*. Moreover, to connect the $u_{i,j}$, we have two options:

1. Take all $v_{i}$ and *y*: With these vertices, we cover the remaining edges and we obtain a CVC of size $(k+2)(u+1)+k+2+2=(k+2)(u+2)+2$. We will name this solution $S_1$ and we will also name $c=(k+2)(u+2)$ making the size of this solution $c+2$. (See Fig. 1)
2. Take a selection of $F_\ell$ that covers all columns and also take $v_{k+2}$ in order to connect the vertex $u_{k+2,0}$. Finally, we also take *y* to cover the edges $(v_i,y)$. This makes a total of $(k+2)(u+1)+SCsol+1+3$ where $SCsol+1$ stands for the size of a Set Cover solution for $({U},{\mathcal {F}})$ together with the vertex *x* and $+~3$ stands for the vertices *f*, $v_{k+2}$ and *y*. We will name this solution $S_2$. (See Fig. 2)

Mixing these two strategies is not a good option because, for any column *j* missed by the set cover, in order to connect every vertex $u_{i,j}$ to the rest of the vertex cover, we would need to take all vertices $v_i$, $1\le i\le k+2$, into the cover, rendering the selection of any $f_{\ell }$ pointless.

Both solutions are of the same size if and only if the size of the optimal set cover for ${\mathcal {F}}$ is *k*.

Given a set cover instance, with $u>k$, then the Connected Vertex Cover instance $((G,c+2),S_1,(G+e,c+1))$ is a valid reoptimization instance for $e^+$-Connected Vertex Cover.

In order to solve the instance for $G+e$, we have to consider that, once the dashed edge is added to the graph, $S_2$ is still a solution and $S_2\setminus \{v_{k+2}\}$ is also a solution. This vertex is not needed anymore because now the edge *e* already connects $u_{k+2,0}$, via the vertex *x*, to the rest of the vertex cover. However, it is easy to see that one cannot remove any vertex of $S_1$. This means that, if the original instance for set cover had a set cover of size *k*, only $S_2\setminus \{v_{k+2}\}$ is optimal, once the new edge *e* is added.

We have just described a polynomial parameter transformation from Set Cover (SC) to $e^{+}$-Connected Vertex Cover. Where an instance $(({U},{\mathcal {F}}, k), u)\in \mathrm{SC}$ if and only if the transformed instance $((G,c+2),S_1,(G+e,c+1))\in e^{+}$-CVC. Thus, the existence of a polynomial reoptimization kernel for $e^{+}$-Connected Vertex Cover would mean that we can a construct polynomial compression for Set Cover. But Set Cover parameterized by the size of the universe does not admit a polynomial compression, unless $\mathrm{NP}\subseteq \mathrm{coNP}/\mathrm{poly}$. $\square$

## Reoptimization and vertex cover

Another case where we observe the power of reoptimization in parameterized problems is in the Vertex Cover (VC) problem. Vertex Cover is a problem in PK, whose best known polynomial kernel is of size 2*k* using linear programming [27]. However, using crown decomposition only allows us to achieve a kernel of size 3*k* in the classical setting [3] (see also the reference book [20]). Recently and independent of our work, Li and Zhu reduced the size of the crown decomposition kernel by refining the method [26]. We present here a way to achieve a reoptimization kernel of size $2k+1$ using crown decomposition. The following proof is not meant to substitute the way that crown decomposition is used in the classical setting, but as a proof of concept of how one might be able to achieve kernelizations for reoptimization problems, based on the solutions given for the neighboring instances.

First we define the problem. A vertex cover of a graph $G=(V,E)$ is a subset $A\subseteq V$ such that every edge is covered, i. e., every edge $e\in E$ is incident to a vertex $v\in A$. As a parameterized problem, we say $(G,k)\in \mathrm{VC}$ if there exists a vertex cover of *G* of size *k* or smaller.

Given a graph $G=(V,E)$, a *matching* *M* is a subset of edges without common vertices, that is, no two edges in a matching can be incident to the same vertex. The vertices that are incident to edges in *M* are called matched vertices; the rest of the vertices in *G* are unmatched.

**Fig. 3:**
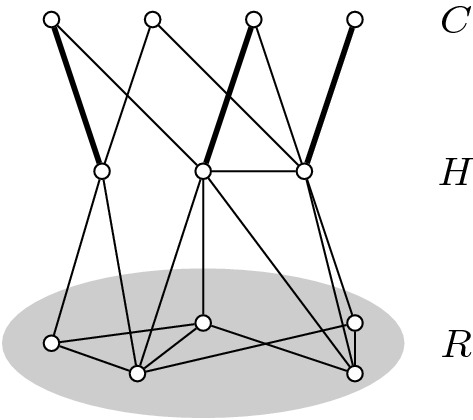
Example of a crown decomposition of a graph

The crown decomposition is a structure in a graph that can be defined as follows. (It is shown schematically in Fig. 3.)

### Definition 10

Let $G=(V,E)$ be a graph. A *crown decomposition* crown decomposition is a partition of *V* into three sets *C*, *H*, and *R* satisfying the following properties.

1. *C* is a non-empty independent set in *G*,
2. There are no edges between *C* and *R*,
3. The set of edges between *C* and *H* contains a matching *M* of size |*H*|; we also say that *M* *saturates* matching.

We call *C* the *crown*, *H* the *head*, and *R* the *rest* of the crown decomposition.

The crown lemma tells us under which conditions crown decompositions exist and is the basis for kernelization using crown decomposition.

### Lemma 1

[3] Let *G* be a graph without isolated vertices and with at least $3k+1$ vertices. There is a polynomial-time algorithm that either finds a matching of size $k+1$ in *G* or finds a crown decomposition of *G*.

This lemma allows us to reduce any Vertex Cover instance to size at most 3*k*. This is because, given a graph of size larger than 3*k*, we either find a matching of size $k+1$ or a crown decomposition of *G*. Given a crown decomposition of *G* into *H*, *C* and *R*, take the maximum matching between *H* and *C*. This matching provides proof that any vertex cover for *G* will need at least |*H*| vertices to cover the edges in the matching. Furthermore, these vertices need to be in *H* to cover all of the edges between *C* and *H*. Thus, we may reduce an instance (*G*, *k*) to an instance $(G-(H\cup C),k-|H|)$.

A *maximal matching* is a matching that cannot be extended. More precisely, a matching *M* is maximal if every other edge $e\in E\setminus M$ is incident to a matched vertex. Observe, that maximal matchings might not have maximum cardinality. On the other hand, a *maximum matching* is a matching with maximum cardinality.

Given a graph $G=(V,E)$ and a matching *M*, an *alternating path* is a path that begins in an unmatched vertex and whose edges belong alternately to $E\setminus M$ and *M*. An alternating path is an *augmenting path* if it begins and ends in unmatched vertices. If one has an augmenting path, one can exchange the edges in the path that are *M* for the edges in the path that are not in *M* and obtain a matching with one more edge.

Let us consider the Vertex Cover problem under edge addition, i. e., $e^+$-VC. Given a vertex cover of size *k* for a graph *G*, we will give a kernel of size 2*k* for $(G+e,k')$ using crown decomposition.

### Theorem 14

Given graph *G*, with a vertex cover $A\subseteq V(G)$ of size *k*. The reoptimization instance $((G,k),A,(G+e,k'))$ for $e^{+}$-Vertex Cover has a polynomial reoptimization kernel of size $2k+1$ constructed using crown decomposition.

### Proof

First of all, let $G=(V,E)$ be a graph, and let $A\subseteq V$ be a vertex cover *G* of size *k*, and $B\subseteq V$ the rest of the vertices in *G*. Thus, $|A|=k$ and $|B|=n-k$. Observe, that there might be edges between vertices of *A* but not between vertices of *B*; otherwise, *A* would not be a vertex cover.

**Fig. 4:**
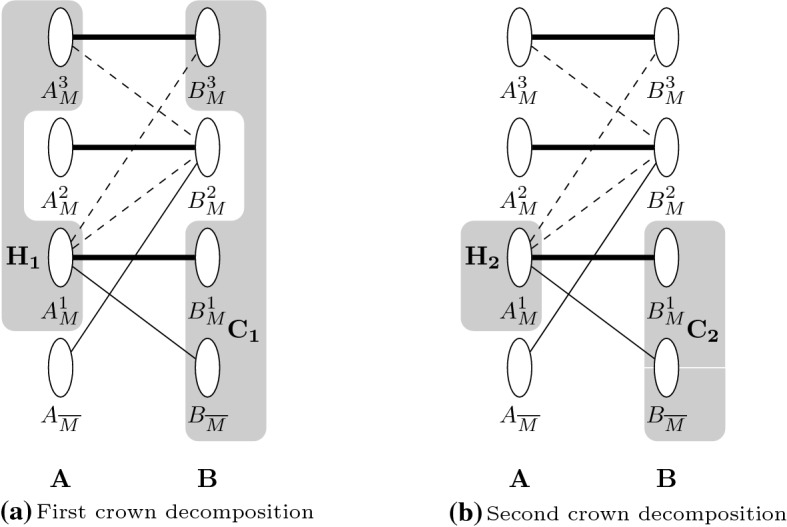
Partition of *G*. Bold edges belong to the matching, solid edges indicate that there exist edges between the two subsets and dashed edges indicate that edges might exist between the two groups. Any non-edge between *A* and *B* indicates that edges do not exist between the two subsets by construction. The shaded subsets indicate the head and crown of two possible crown decompositions parting from this partition of *G*; the non-shaded subsets are the rest sets $R_1$ and $R_2$, respectively

Let us pick *M* to be a maximum matching between *A* and *B*. We will now partition *A* and *B* further into subsets according to their adjacencies (see Fig. 4). First, we consider the vertices of *A* and *B* that are not part of the matching, let us call these vertex sets $A_{{\overline{M}}}$ and $B_{{\overline{M}}}$, respectively. The vertices of these two subsets do not share edges because otherwise *M* would not be maximal. The vertices of *A* and *B* that are part of the matching will be $A_{M}$ and $B_{M}$, respectively. Now let $A_{M}^1$ be the matched vertices in *A* that have an alternating path to at least one vertex in $B_{{\overline{M}}}$, and let $B_{M}^1$ be the vertices matched to those from $A_{M}^1$. Let then $B_{M}^2$ be the vertices in *B* that have an alternating path to at least one vertex in $A_{{\overline{M}}}$, and let $A_{M}^2$ be the vertices matched to those from $B_{M}^2$. These four subsets have no intersection because otherwise there would be an augmenting path starting in $B_{{\overline{M}}}$ through an alternating path to $v\in A_{M}^1\cap A_{M}^2$ and through its matched vertex in $B_{M}^2$ and another alternating path to $A_{{\overline{M}}}$, contradicting the fact that *M* is a maximum matching. Let then $A_{M}^3$ and $B_{M}^3$ be the rest of the matched vertices in *A* and *B*, respectively. Neither $B_{{\overline{M}}}$ nor $B_M^1$ have edges to $A^3_{M}$ by construction. In the first case, vertices in $A_M$ with edges to $B_{{\overline{M}}}$ are placed in $A^1_M$. In the second case, any edge from $B_M^1$ to a vertex $v\in A^3_M$ would imply the existence of an alternating path starting at $B_{{\overline{M}}}$ to $A^1_{M}$ and through the matching to $B^1_M$, through the existing edge from $B_M^1$ to *v*, such an alternating path would mean, by definition that $v\in A^1_M$.

Observe, through Fig. 4a, that the following is a valid crown decomposition for *G*:

$$\begin{aligned}B_{{\overline{M}}}\cup B_{M}^1 \cup B_{M}^3=C_1\text {, }A_M^1\cup A_M^3=H_1\text { and }A_{{\overline{M}}}\cup A_M^2\cup B_M^2=R_1.\end{aligned}$$

This is true because *B* is an independent set by construction, and there are no edges between $C_1$ and $R_1$ because $B_{{\overline{M}}}$ and $B_{M}^1$ have edges neither to $A_{{\overline{M}}}$ nor to $A_M^2$ and neither does $B_{M}^3$. Moreover, as depicted in Fig. 4b,

$$\begin{aligned}B_{{\overline{M}}}\cup B_{M}^1=C_2\text {, }A_M^1=H_2\text { and }B_{M}^3\cup A_{M}^3\cup R_1=R_2,\end{aligned}$$

is also a valid crown decomposition, as $B_{{\overline{M}}}$ and $B_{M}^1$ also have no edges to $A_{M}^3$.

In the second crown decomposition, $|R_2|\le 2k$ because $|A|\le k$ and thus $|B_M|\le k$, meaning that $|B_{{\overline{M}}}|\ge n-2k$ and $B_{{\overline{M}}}$ is always part of *C*. If $H_2$ is empty, it means that there is a set of isolated vertices in *G* of size at least $n-2k$ and we can erase them. Thus, except in the special case where $H_2$ is empty, $|C_2|\ge n-2k+1$, $|H_2|\ge 1$, and $|R_2|\le 2k-1$.

First, we observe that if, after adding the new edge *e* the maximum matching *M* can be extended to a maximum matching of size $k+1$, then no vertex cover can exist of size *k* or smaller; thus, if $k'\le k$ this would be a no answer. Moreover, if $k'>k$, covering the new edge would only require adding at most one more vertex in the cover, which can be trivially done by extending *A* and not exceed the new value of the parameter.

Now assume that $k'\le k$ and the maximum matching *M* cannot be extended by using the new edge *e*. We prove using these two crown decompositions that we can construct a kernel of size $2k+1$ for any $G+e$ under edge addition.

If the new edge is incident to an isolated vertex, we can use the following reduction: Observe that $G+e-\{\text {isolated vertices}\}$ contains a leaf, i.e., a vertex of degree 1, in particular the formerly isolated vertex. Add the vertex adjacent to the leaf to the cover and use $R_2$ of the second crown decomposition as a 2*k*-sized kernel.

If the new edge *e* is adjacent to any vertex in *A* and $k'=k$, the vertex cover for *G* is also a vertex cover for $G+e$, and thus, the problem is solved. If $k'< k$ and *e* is adjacent to *A*, we can adapt the further partition of *A* and *B* to take into account the new edge *e* extending the maximum matching if necessary and obtain, via the first or second crown decompositions a kernel of size 2*k* consisting of the rest. Observe that this new edge adjacent to *A* cannot reduce the size of the maximum matching, only increase it, thus, the size of the rest can only decrease.

If *e* is adjacent to two vertices *u* and *v* in *B*, we make the following case distinction.

$$\mathbf{Case 1}$$
$u,v\in B^2_{M}\cup B^3_{M}$: $C_2,\ H_2$ and $R_2$ are also a crown decomposition for $G+e$.
$$\mathbf{Case 2}$$
$u,v\in B_{{\overline{M}}}$: Set $H=H_1\cup u$ and $C=C_1-u$ and $R=R_1$. The new edge *e* provides the matching between *u* and *v*, so *H*, *C* and *R* as defined are a crown decomposition.
$$\mathbf{Case 3}$$
$u\in B_{{\overline{M}}}\text {, }v\in B^2_{M}\cup B^3_{M}$: Set $R=R_2\cup u$, $C=C_2-u$ and $H=H_2$. This provides a valid crown decomposition, as *e* will be left inside *R*. Observe, that because $R_2<2k$, $R\le 2k$.
$$\mathbf{Case 4}$$
$u\in B^1_{M}\text {, }v\in B^2_{M}\cup B^3_{M}$: There is always an alternating path between the vertex matched to *u* and $B_{{\overline{M}}}$. This path provides an alternative maximum matching $M'$ that does not use *u*. Thus, we are in Case 3.
$$\mathbf{Case 5}$$
$u\in B^1_{M}\text {, }v\in B^1_{M}\cup B_{{\overline{M}}}$: Using the same technique as in Case 4, we can assume $v\in B_{{\overline{M}}}$. If there is an alternating path from *u* to $B_{{\overline{M}}}-v$, we can, again, use the same technique as in Case 4 and we are in Case 2. Otherwise, every alternating path from *u* to $B_{{\overline{M}}}$ leads exclusively to *v*. Meaning that there is a set of vertices $B_v\subseteq B^1_M$ and $A_v\subseteq A^1_M$ that do not contain edges to any other vertex in $B^1_M$ or $B_{{\overline{M}}}$. Redefining $R= v\cup B_v\cup A_v\cup R_1$, $H=H_1-A_v$ and $C=C_1-(B_v\cup v)$, we have a valid crown decomposition. Observe that the only unmatched vertices in *R* are $A_{{\overline{M}}}$ and *v*, thus, if $A_{{\overline{M}}}$ is not empty, $|R|\le 2k$, but if $A_{{\overline{M}}}$ is empty and $A_v$ contains all of *A*, $|R|\le 2k+1$.

For every crown decomposition we defined, the set *R* contains not more than $2k+1$ vertices; thus, these decompositions provide a kernel of size $2k+1$ for $e^+$-Vertex Cover. $\square$

If we consider Vertex Cover with other local modifications, we observe it is easy to use the same technique. Adding vertices and deleting vertices or edges allow us to use exactly the same crown decomposition and similar techniques to find kernels of size at most $2k+1$.

## Conclusions and further research

We presented examples of problems that do not have polynomial kernels under standard complexity-theoretic assumptions, but whose reoptimization versions have polynomial reoptimization kernels. We also presented an example, where a kernel using the same crown decomposition technique is smaller in the reoptimization version of the problem than in the original classical version. We finally presented a reduction proving that there are problems and local modifications, for which the complexity does not decrease when considering reoptimization.

In conclusion, there are problems that are easier under reoptimization conditions and problems that are not. We hope that further research will help us to better understand how much information neighboring solutions are providing and when this information is helpful.

The relation between Turing kernelization and reoptimization kernels is still unclear and can be a topic for further research. In another direction, one can try to obtain improved polynomial kernels for parameterized problems through reoptimization kernels.
